# Form and function of the apical extracellular matrix: new insights from *Caenorhabditis elegans, Drosophila melanogaster*, and the vertebrate inner ear

**DOI:** 10.12703/r/9-27

**Published:** 2020-12-22

**Authors:** Sherry Li Zheng, Jennifer Gotenstein Adams, Andrew D Chisholm

**Affiliations:** 1 Department of Developmental Biology, Stanford University School of Medicine, Stanford, CA 94305, USA; 2 Section of Cell and Developmental Biology, Division of Biological Sciences, University of California San Diego, La Jolla, CA 92093, USA

**Keywords:** epithelia, collagens, zona pellucida domain, chitin, tubulogenesis

## Abstract

Apical extracellular matrices (aECMs) are the extracellular layers on the apical sides of epithelia. aECMs form the outer layer of the skin in most animals and line the luminal surface of internal tubular epithelia. Compared to the more conserved basal ECMs (basement membranes), aECMs are highly diverse between tissues and between organisms and have been more challenging to understand at mechanistic levels. Studies in several genetic model organisms are revealing new insights into aECM composition, biogenesis, and function and have begun to illuminate common principles and themes of aECM organization. There is emerging evidence that, in addition to mechanical or structural roles, aECMs can participate in reciprocal signaling with associated epithelia and other cell types. Studies are also revealing mechanisms underlying the intricate nanopatterns exhibited by many aECMs. In this review, we highlight recent findings from well-studied model systems, including the external cuticle and ductal aECMs of *Caenorhabditis elegans, Drosophila melanogaster*, and other insects and the internal aECMs of the vertebrate inner ear.

## Introduction

The extracellular matrix (ECM) is the network of collagens, proteoglycans, lipids, enzymes, and other components that surround cells. These networks provide structural and biochemical support to cells and tissues and are involved in the communication between a cell and its external environment, including surrounding cells. The ECM plays critical roles in many developmental processes, including tissue homeostasis, differentiation, and morphogenesis in all animals^1^. ECMs can be classified as apical, basal, or stromal. Basal ECMs, also known as basal laminae or basement membranes, are universal and ancient features of animal epithelia^2^ and contain conserved core proteins such as laminins and type IV collagens. In contrast to basal and stromal ECMs, which serve specialized functions on the interior sides of tissues, apical ECMs (aECMs) act as barriers along the outer surfaces of epithelial tissues such as the skin or internal tubular organs. External aECMs, such as the cuticle of nematodes and arthropods, are highly specialized for their ecological niches yet share common features such as regulation by matrix crosslinking enzymes. External aECMs can incorporate lipid layers that provide desiccation resistance and permeability barrier function. Internal aECMs, such as those of tubular epithelia, often have transient developmental roles in shaping tube morphology^3,4^. Other aECMs found in vertebrates include the vascular glycocalyx, the mucin-rich coating of the gastrointestinal tract and upper airways, and the alveolar surfactant of lungs. Compared to the conserved basal ECM, aECMs are diverse in composition and function but contain major conserved families such as collagens, lectins, and mucin-related glycoproteins (Table 1). Proteins with zona pellucida (ZP) domains^5^ are associated with early stages of aECM formation in multiple model systems, while the rigidity of external aECMs is often provided by layers of the amino polysaccharide chitin. Here we review recent advances in understanding the biosynthesis, patterning, and signaling roles of selected model aECMs.

**Table 1.** Selected proteins of the apical extracellular matrix.

Protein family or role	*Caenorhabditis* *elegans*	*Drosophila melanogaster*	Vertebrates	References
Collagens	COL-182 BLI-1		Collagens II, V, IX, and XI	14,23 2
Collagen processing	TMEM-131			13
Transcription factors	LIN-29 ELT-3	Blimp-1 Yorkie (YAP/TAZ) Ichor Ubx		23,24 25–28
ZP domains	FBN-1 NOAH-1 NOAH-2	Dumpy (Dp) Piopio (Pio) NompA Trynity (Tyn)	α-tectorin (TECTA) β-tectorin (TECTB)	15,16 3,12,29 30,31
Nidogen + EGF	DEX-1		α-tectorin (TECTA)	12,19 30,31
eLRRon	LET-4, EGG-6, SYM-1	Artichoke		17 32
Extracellular signaling pathways	DAF-7/TGFβ DBL-1/BMP NPR-8			33–36
ABC transporters + related		Snustorr (Snu) Oskyddad (Osy) Snu-like (Snsl)	ABCA12	37,38 39
Osiris		Gore-tex (Gox)		40
Chitin biosynthesis/ deposition		Obstructor-A (ObstA) Expansion (Exp) Rebuff (Reb) Apnoia (Apn)		3,41–43
C-type lectin		Schlaff (Slf)		44
Crosslinking enzymes		Alas		26
Proteases		Lumens interrupted (Lint) Notopleural (Np) Tracheal-prostasin (Tpr)		27,45
Glycoproteins			Otogelin (OTOG) Otogelin-like (OTOGL) Otoancorin Stereocilin	30,46

Only selected proteins are shown owing to limitations of space.

## *Caenorhabditis elegans* aECM components and biosynthesis

**Figure 1.**
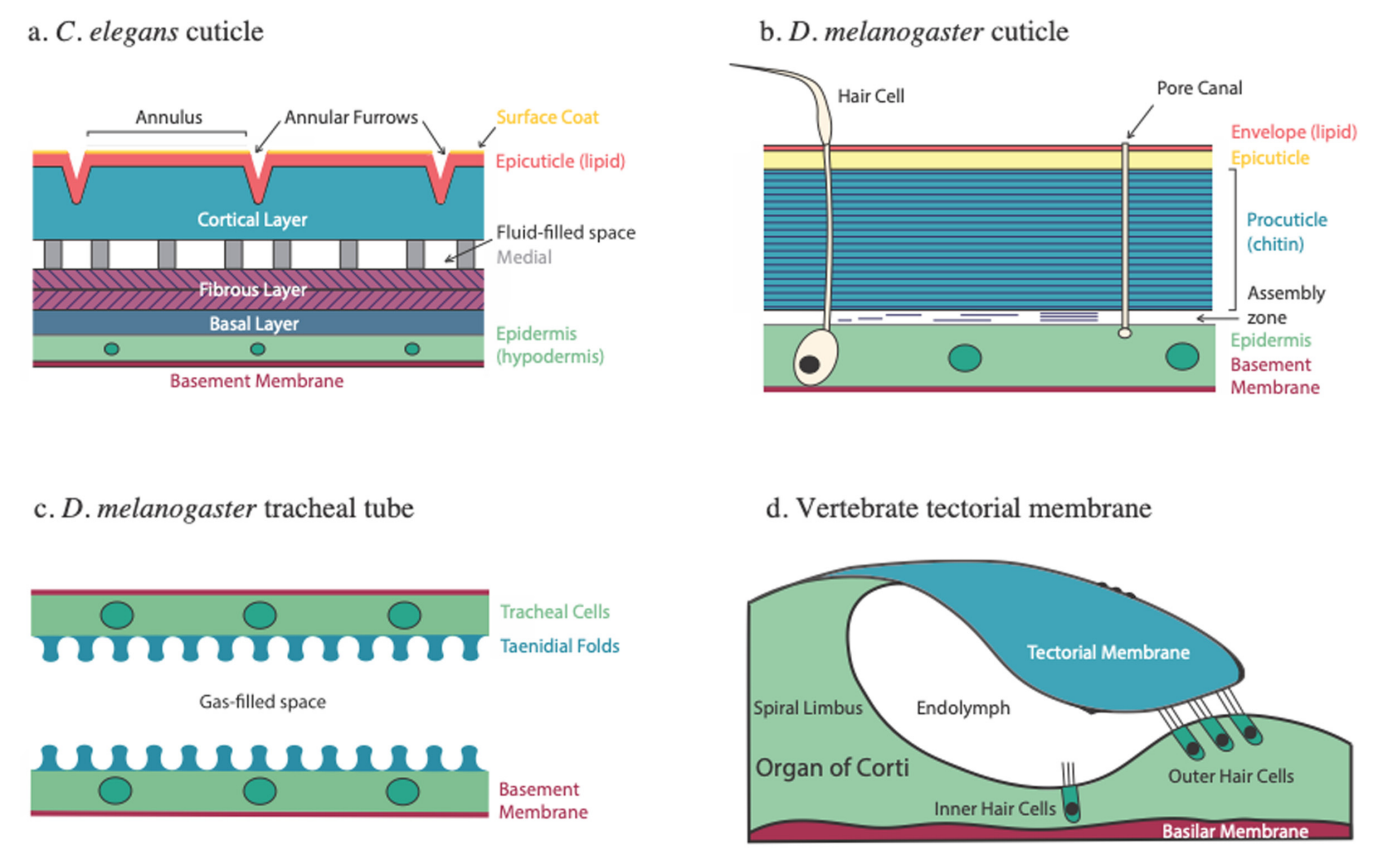
Apical extracellular matrices of *Caenorhabditis elegans*, *Drosophila melanogaster*, and the vertebrate inner ear. **a**. Cartoon of adult *C. elegans* cuticle, longitudinal section. The cuticle consists of an outer surface coat (gold), a lipid-rich epicuticle (red), collagenous cuticle proper (divided into cortical [blue], medial [gray], fibrous [purple], and basal [navy] layers). The cuticle is secreted by the underlying cellular epidermis (green), internal to which is a basement membrane (burgundy). **b**. Cartoon of *D. melanogaster* cuticle, consisting of an outer lipid-rich envelope (red), glycoprotein-rich epicuticle (yellow), and the chitinous procuticle (blue), formed in the assembly zone at the apical surface of the underlying epidermis (green). Pore canals form channels through the cuticle layers to the surface. **c**. Cartoon of *D. melanogaster* mature tracheal cuticle, showing taenidial folds (blue). Envelope and epicuticle are not shown for simplicity. **d**. The vertebrate tectorial membrane (blue) is shown in cross section, attached to the underlying epithelial ridge of the organ of Corti at its medial edge and to the stereocilia of the outer hair cells at its lateral edge. The underlying basement membrane is known as the basilar membrane.

As the outermost layer of *C. elegans* skin, the cuticle is an accessible model for studies of aECMs. The *C. elegans* epidermis (known as the hypodermis) secretes a complex multilayered aECM beginning in late embryogenesis and thereafter in four molts, resulting in the mature adult cuticle consisting of an outer surface coat, a lipid-rich epicuticle, and the multilayered collagenous cuticle (Figure 1a)^6–8^. Other epithelial aECMs in *C. elegans* include those of internal tubes, such as the excretory duct and vulva, and specialized aECMs of sensory organs^4,9^. Recent bioinformatic analysis has enumerated components of the *C. elegans* ECM (including aECMs), predicting a total “matrisome” of over 700 proteins^10^. The 43 *C. elegans* ZP proteins have been further classified by phylogenetic methods^11,12^. This “parts list” for *C. elegans* aECMs remains dauntingly complex, with functional or expression data lacking for many proteins.

One longstanding question remains: how do complex aECM structures assemble, starting in the secretory pathway and concluding in the extracellular space? Elegant genetic screens identified the conserved protein family TMEM131 as being important for ER processing of cuticle collagen cargos^13^; TMEM131 may directly interact with collagens. Interestingly, TMEM131 function in collagen biosynthesis appears to be conserved, despite low sequence conservation between nematode cuticle collagens and collagens in other organisms. Despite their molecular diversity, aECMs may share common factors in their biogenesis.

Just over 10% of the 180 *C. elegans* cuticle collagens were identified in classical phenotype-based screening. Loss or gain of function in certain cuticle collagens can have dramatic effects on body morphology, leading to phenotypes such as dumpy (Dpy), long (Lon), roller (Rol), or blistered (Bli); the lack of functional information on other collagens may reflect genetic redundancy. A recent report found that the laboratory “wild-type” strain (N2) accumulated a loss-of-function mutation in the collagen *col-182* that affects the phenotypes caused by other collagen mutations^14^. The standard wild-type strain itself may be sensitized to alterations in cuticle function compared to the ancestral *C. elegans*.

The first embryonic epidermal aECM, known as the pre-cuticle or embryonic sheath, precedes the collagenous cuticle and is essential for epidermal morphogenesis. The pre-cuticle contains multiple ZP-domain proteins including FBN-1^15^, NOAH-1, and NOAH-2^16^, as well as extracellular leucine-rich repeat proteins^17^ and lipocalins^18^. The nidogen- and EGF-domain transmembrane protein DEX-1 localizes to multiple aECMs; *dex-1* loss-of-function mutants resemble multiple ZP mutants, suggesting that DEX-1 may act together with one or more ZP proteins^12^. DEX-1 itself is upregulated in response to starvation and is required for structural and functional remodeling of the cuticle during the dauer stage^19^. Subsets of ZP-domain proteins or their interacting partners may contribute to the unique characteristics of other aECMs, such as those of the excretory canal lumen, sensory organs, or the vulva lumen^9,20^. Interestingly, many of these aECMs play transient developmental roles in the initial establishment of an aECM.

Comparatively little remains known of the biogenesis or function of the lipid-rich epicuticle, which shares some ultrastructural similarities with lipid bilayers. Alterations in surface lipid composition have been linked to defects in cuticle integrity^21^ and resistance to surface bacterial infections^22^. In *C. elegans*, lipid-binding lipocalins LPR-1 and LPR-3 are required for normal surface lipid organization and may play roles in transport or localization of aECM lipids to the epicuticle^18^.

## The aECM as a signaling center: interactions between the *C. elegans* cuticle and other cells

Emerging evidence has revealed complex interactions between the cuticle and the epidermis. Many cuticle collagens are upregulated during the synthesis of new cuticle during embryogenesis and/or the four post-embryonic molts; within each molt, collagen transcription is precisely timed (within 1–2 hours) to coincide with the cuticle layer being secreted^47^. Cuticle collagens are also regulated by transcriptional pathways that control the developmental progression of stages; for example, multiple collagens are activated by the zinc finger transcription factor LIN-29, which coordinates the larval-to-adult switch. LIN-29 and its collagen targets are also upregulated in response to loss of function in the collagen BLI-1, a model of cuticle damage^23^, suggesting that collagen expression by the epidermis is subject to feedback regulation to maintain cuticle integrity. Disruption of collagens localized to annular furrows triggers specific stress or antimicrobial responses, suggesting furrows are required for sensing environmental conditions^48^. Conversely, cuticle composition may be regulated by environmental conditions such as nutrition or population density, possibly via the epidermal GATA factor ELT-3^24^.

Molting involves the loss of a cuticle layer and its replacement by a new, distinct layer. New findings suggest that the disruption of contacts between the epidermis and cuticle may trigger transcriptional activation of epidermal lysosome function^49^. “Activated” lysosomes appear to degrade components of the old cuticle, possibly generating raw materials for fresh cuticle synthesis. Loss of function in specific cuticle collagens increases autophagy in the epidermis, dependent on the ciliary transport function of ASI sensory neurons and the TGFβ signal DAF-7^33^. Ciliated sensory neurons contact parts of the epidermis and cuticle and might sense cuticle integrity directly or indirectly. These studies also suggest overlap between molting pathways and factors involved in damage sensing in the mature epidermis.

The integrity or function of the cuticle may also be sensed by other tissues, implying complex systemic regulation of cuticle composition and function. For example, altered collagen levels can affect the level of the neuronally expressed BMP signal DBL-1^34^, which acts via SMADs in the epidermis to regulate cuticle collagen expression^35^. These observations suggest the operation of a feedback pathway that senses cuticle function; the precise mechanism of such feedback is not yet clear but could involve mechanosensing by neurons. Cuticle collagen synthesis is also inhibited by the neuronally expressed neuropeptide receptor NPR-8, leading to elevated *Pseudomonas* resistance of *npr-8* mutants^36^. How neuronal NPR-8 affects collagen expression also remains to be determined.

## The aECM of the *Drosophila melanogaster* cuticle and trachea

Like the nematode cuticle, the *D. melanogaster* cuticle is layered and serves a protective role but is more rigid and highly pigmented; *D. melanogaster* also produces specialized aECMs for sensory organs, appendages, and organ tube formation. The *D. melanogaster* cuticle, like that of most insects, contains three major layers: the lipid-rich envelope, protein-rich epicuticle, and a chitinous procuticle (Figure 1b). The procuticle provides mechanical strength, while the envelope has a barrier function.

Recent work has shed light on the formation of the lipid-rich envelope. The ZP-domain protein Trynity (Tyn) is required for epidermal barrier function; ultrastructural analysis indicates Tyn is specifically required for the lipid envelope^29^. The envelope also contains cuticulin, a complex waterproof molecule composed of protein, lipid, and catecholamine. Cuticulin deposition requires nanopore canals connecting the apical epidermis to the exterior cuticle. The ABC transporter Snustorr (Snu) localizes to the apical plasma membrane and transport vesicles and is required for cuticulin and lipid deposition to the envelope^37^. The secreted Snu-like protein (Snsl) is also required for proper cuticulin deposition and localizes to pore canals, dependent on Snu^37^. Another ABC transporter, Oskyddad (Osy), localizes to pore canals and is required for transport of cuticular hydrocarbons to the cuticle surface^38^. Unlike Snu, Osy mutants do not affect envelope integrity, and Osy and Snu independently localize, suggesting that Osy and Snu act in parallel to establish the envelope barrier^38^. ABC transporters such as ABCA12 have also been implicated in the biogenesis of lipid-rich components of vertebrate aECM such as keratinocyte lamellar bodies and pulmonary surfactant, suggesting possible molecular similarities in extracellular lipid-based aECM formation^39^.

Nanopores in the cuticle of olfactory sensilla allow odorants to reach neurons while blocking large molecules and preventing significant fluid loss^40^. Ultrastructural analysis indicates that cells initially form electron-dense protrusions (plasma membrane plaques) which provide a scaffold for the assembly of cuticle envelope from envelope segments, thereby forming a nanopore. The Osiris family transmembrane protein Gore-tex (Gox) localizes to intracellular vesicles during cuticle secretion and may regulate trafficking of envelope components or patterning of endocytic or exocytic activity^40^.

Cuticle assembly occurs at the epidermal apical plasma membrane in the “assembly zone” (Figure 1b), where chitin fibers are organized into sheets (laminae) by enzymes and structural proteins including chitin synthase, chitin deacetylases, chitin-associated proteins (e.g. Obstructor-A/ObstA), ZP-domain proteins (Dumpy/Dp and Piopio/Pio), and chitinases^3^. Stabilization of the cuticle depends on crosslinks mediated by catecholamines, glutamine–lysine bridges, and dityrosines. The C-type lectin Schlaff (Slf) is required for cuticle compactness and appears to promote dityrosine-mediated adhesion between the epicuticle and procuticle layers^44^. Studies in other insects are also revealing how the fine structure of the chitinous aECM may be regulated. In the locust *Locusta migratoria,* chitin orientation is under circadian control, with unidirectional fibers forming during the day and helicoidal (left-twisting) fibers at night^50^. Day microvilli are oriented such that chitin fibers are secreted into the assembly zone in their final orientation, whereas night fibers are secreted then self-assembled into the final helicoidal structure^51^. This work suggests that fine aECM structure may be determined by the regulated interplay of templating on cellular structures and extracellular self-assembly mechanisms.

## Tracheal tube morphogenesis and the *D. melanogaster* **aECM**

Respiration in *D. melanogaster* is mediated by tracheal tubes, which extend from the cuticle and ramify through the body. The aECM of the trachea forms in two phases: first, a central chitin filament forms along the tracheal lumen and promotes tube expansion at the expense of tube elongation, then this filament is degraded and cuticle ridges (taenidial folds) (Figure 1c) form in a supracellular helix that extends along the trachea. The regular spacing of these taenidial folds is thought to involve self-organizing processes in the cortical actin cytoskeleton of the tracheal epithelium^52^ as well as feedback regulation between the cytoskeleton and the aECM^53^. As tracheal aECM has been extensively reviewed^3,53,54^, we highlight selected recent insights.

Multiple transcription factors regulate the expression of aECM components and enzymes for normal tracheal tube morphogenesis, and, as in *C. elegans*, some appear to be under feedback control by cuticle integrity or function. Multiple aECM regulators are repressed by Blimp-1^25^, whose activity may be regulated by the feedback mechanism that regulates taenidial spacing^53^. The Hippo pathway transcription factor Yorkie (YAP/TAZ) has a dual role in tracheal morphogenesis, promoting tube elongation via the expression of cytoskeletal factors, and promoting water tightness via expression of the crosslinking enzyme Alas^26^. YAP/TAZ signaling may coordinate the regulation of tracheal cytoskeletal dynamics and the aECM. Finally, the zinc finger protein Ichor is required for correct lumen shaping in the seamless tubes of terminal tracheal branches^27^ and promotes the transcription of multiple aECM regulators or components, including members of the Osiris family and the serine protease Lumens Interrupted (Lint)^27^.

Tracheal chitin deposition requires chitin synthase and two related proteins, Expansion (Exp) and Rebuff (Reb)^41^. *Reb* mutants display slightly reduced luminal chitin and severely reduced luminal chitin-binding protein ObstA, leading to overall reduced aECM^42^. Reb and Exp contain MH2 domains also found in SMAD proteins but are thought to act independently of canonical TGFβ signaling; Reb may be required for normal endocytosis or recycling of ObstA or other components^42^. Apnoia (Apn), a novel transmembrane protein localized to the apical side of the tracheal lumen, is required for normal tube length and aECM deposition and may be involved in vesicular trafficking of tracheal aECM^43^. Tracheal aECM is degraded and removed in a coordinated wave of endocytosis^55^, but how aECM is rapidly cleared from the lumen remains to be fully understood. Pore-like taenidial channels between the taenidial folds likely control the passage of aECM to the apical plasma membrane for endocytosis^29^. As well as having defective epidermal barrier function, *Tyn* mutants display aberrant taenidial channels, likely impairing endocytic clearance of luminal aECMs and resulting in defective gas filling of the trachea^29^. The matriptase-related protease Notopleural (Np) is also essential for liquid clearance and gas filling of the tracheal tubes^45^. *Np* mutant embryos have normal chitin deposition and clearance but develop an unstructured aECM lacking taenidial folds. *Np* mutants deposit Dp in the aECM but are blocked in Dp condensation or degradation^45^. Targets of Np cleavage include the protease tracheal-prostasin (tpr) and another ZP protein, Pio^45^, suggesting conservation of the matriptase–prostasin proteolytic cascade between vertebrates and *D. melanogaster.*

## *D. melanogaster* appendage morphogenesis involves dynamic regulation of the aECM

Recent findings highlight the critical role aECMs play in morphogenesis in external appendages such as legs, wings, and halteres. Epithelial cells undergo three-dimensional shape changes to drive morphogenetic cell height expansion of the apical surface of the *D. melanogaster* wing and leg^56^. The protease Stubble (Sb) mediates degradation of aECM components such as the ZP-domain protein Dp, allowing convergent extension of the underlying epithelium and wing elongation^56^. Evolutionary changes in appendage morphology may also reflect changes in aECM function: modern-day two-winged flies evolved from four-winged ancestors, with the vestigial posterior wings now functioning as balancing organs (halteres). The HOX transcription factor Ultrabithorax (Ubx) represses wing development and promotes haltere development, in part by repression of the aECM proteases Sb and Np, resulting in failure to degrade Dp and thereby preventing aECM remodeling and wing extension^28^. Development of another morphological novelty, the posterior lobe of *D. melanogaster* male genitalia, is also regulated by expanded expression of the aECM component Dp, revealing variation in aECM expression as a pathway of morphogenetic evolution^57^.

## Patterning the aECM of the vertebrate inner ear

The function of the mammalian inner ear rests on a set of mechanosensory epithelia and associated aECMs that form highly organized 3D structures known in mammals as the cupula, the otoconial membrane, and the tectorial membrane (TM) (Figure 1d)^58^. The TM is an extended ribbon-like aECM overlying the sensory epithelium of the cochlea (the spiral organ of Corti). It is the most complex inner ear aECM and plays multiple roles in sensory transduction. Much knowledge of inner ear aECMs has come from genetic studies of deafness or other inner ear disorders in humans, mice, or zebrafish^59,60^. These and other studies identified key components of the inner ear aECMs, including collagens (primarily types II, V, IX, and XI) and non-collagenous glycoproteins, including the secreted mucin-like otogelins and the ZP-domain-containing α- and β-tectorins^30^. Mutations in α-tectorin are a common cause of non-syndromic hearing loss, displaying dominant or recessive effects depending on the type of mutation and domain affected.

The TM forms a “roof” between ridges of the sensory epithelium (see Figure 1d). At its medial edge, the TM is anchored to the apical surface of the epithelium via the GPI-anchored protein otoancorin; at its lower lateral edge, the TM contacts the apical stereocilia of three rows of sensorimotor outer hair cells (OHCs). Recent genetic and morphological analysis has revealed that otogelin, otogelin-like, and the membrane-anchored stereocilin play interrelated roles in connecting the tips of OHC stereocilia to the TM^46^. While otogelin, otogelin-like, and stereocilin all localize independently within OHC hair bundles, loss of any one protein disrupts the distribution of the other two, suggesting interdependent roles in TM-OHC attachment.

The TM has been thought to have a primarily mechanical role, both by virtue of its direct connection to OHC stereocilia and via effects on fluid flow in the endolymph of the cochlea^61^. Recent evidence suggests the TM may also regulate the ionic environment surrounding the stereocilia. Fluorescence imaging revealed that the TM had elevated levels of Ca^2+^ compared to surrounding endolymph; loud sounds decrease the concentration of TM Ca^2+^, reducing auditory sensitivity^62^. It is not yet known how the TM regulates Ca^2+^; however, this study suggests that aECMs might have additional unappreciated roles in extracellular calcium flux.

Ultrastructural analysis has revealed the remarkably complex spatial organization of the inner ear aECMs. The mature TM contains multiple structurally distinct layers and regions^63^ as well as other less well-understood structures formed transiently during development^64^. Recent ultrastructural analyses of otoconia or otoliths have also yielded insights into how the aECM regulates biomineralization^65^ and are revealing structural changes in the TM during aging and age-related hearing loss^66^.

The TM in mice develops gradually in late embryogenesis and early postnatal development; in its early stages, the TM is largely composed of the α- and β-tectorins, followed by oriented collagen fibrils^67^. Orientation of the collagen fibrils in the various TM layers is controlled by signals from the epithelium, as planar cell polarity defects in the epithelium disrupt collagen fibril organization^67^. The fine structure of the TM has been thought to arise largely from self-organization in the extracellular space, but recent work suggests that both the membrane-anchored and the secreted forms of α-tectorin are required for organization of the TM^31^. α-tectorin is GPI anchored and recruits collagen fibrils that support the formation of the TM at the cell surface. Subsequent release of α-tectorin by cleavage of the GPI anchor allows the organization of collagens into a multilayered TM, suggesting a model in which the TM develops by “3D printing” of successive layers on the cell surface concomitant with release of the previously patterned layer.

## Remaining questions and future directions

In summary, recent studies have expanded our understanding of aECM biogenesis and function across multiple model organisms and have begun to highlight shared structural and functional principles. With a comprehensive aECM parts list in hand, a clear future goal will be systematic analysis of expression, along the lines of the basement membrane toolkit recently generated for *C. elegans*^68^. In comparison to the protein components of aECM, a key knowledge gap remains the composition and assembly of extracellular lipid layers such as the epicuticle and envelope. High-resolution analytical approaches such as secondary ion mass spectrometry (SIMS) may offer fresh ways to address this problem^69^. Analysis of aECM assembly continues to rely on *in vivo* studies, although a long-term goal remains the development of *in vitro* reconstitution assays for aECM factors. Such assays could generate a better understanding of the interplay between cell-based templating and self-organization in aECM patterning. Improved mechanistic understanding of aECMs could shed light on the complexities of the genotype–phenotype interaction in studies of hearing loss related to TM dysfunction. Finally, although recent work has begun to reveal signaling between aECMs and internal tissues, more studies will be required to understand these pathways at mechanistic levels.
